# A retrospective descriptive study of cranioplasty failure rates and contributing factors in novel 3D printed calcium phosphate implants compared to traditional materials

**DOI:** 10.1186/s41205-020-00066-5

**Published:** 2020-06-17

**Authors:** Michael Koller, Daniel Rafter, Gillian Shok, Sean Murphy, Sheena Kiaei, Uzma Samadani

**Affiliations:** 1 Department of Bioinformatics and Computational Biology, University of Minnesota, 101 Pleasant Street Southeast, Minneapolis, MN 55455 USA; 2 Department of Neurosurgery, Minneapolis VA Medical Center, 1 Veterans Drive, Minneapolis, MN 55417 USA

**Keywords:** 3D-printed, Cranioplasty, Cranial implant, Craniectomy, Traumatic brain injury

## Abstract

**Background:**

Failure rates with cranioplasty procedures have driven efforts to improve graft material and reduce reoperation. One promising allograft source is a 3D-printed titanium mesh with calcium phosphate filler. This study evaluated failure rates and pertinent characteristics of these novel 3D-grafts compared to traditional materials.

**Methods:**

Sixty patients were retrospectively identified who underwent a cranioplasty between January 2015–December 2017. Specific data points related to graft failure were collected for all surgical admissions, from the primary injury to their most recent. These included, but were not limited to, initial physical exam findings, vitals, comorbid conditions, surgery length, estimated blood loss, incision type, and need for revision. Failure rates of 3D-printed allografts were compared to traditional grafts.

**Results:**

A total of 60 subjects were identified who underwent 71 unique cranioplasty procedures (3D = 13, Synthetic = 12, Autologous = 46). There were 14 total failures, demonstrating a 19.7% overall failure rate. Specifically, 15.4% (*n* = 2) of 3D, 19.6% (*n* = 9) of autologous, and 25.0% (*n* = 3) of synthetic grafts required revision. Patients receiving 3D-grafts had the shortest overall mean surgery times (200.8 ± 54.3 min) and lowest infection rates (7.7%) compared to autologous (210.5 ± 47.9 min | 25.0%) and synthetic models (217.6 ± 77.3 min | 8.7%), though significance was unable to be determined. Tobacco use and trap-door incisions were associated with increased failure rates relative to straight or curved incisions in autologous grafts. Cranioplasties performed less than 3 months after craniectomy appeared to fail more often than those performed at least three months after craniectomy, for the synthetic group.

**Conclusion:**

We concluded that 3D-printed cranioplasty grafts may lead to lower failure rates and shorter surgery times compared to traditional cranioplasty materials in our limited population. 3D-implants hold promise for cranial reconstruction after TBI.

## Background

Decompressive craniectomy (DC) is a common neurosurgical intervention in which a large section of the skull is removed in the setting of severe traumatic brain injury (TBI). The resulting skull defect is left open to allow brain tissue to swell past this rigid border, thus mitigating potentially fatal elevations in intracranial pressure [1–3]. Once the underlying pathology has been corrected, the contour of the skull is reconstructed either with the autologous bone flap or a synthetic implant via cranioplasty. This is done for cosmesis as well as to reduce complications from DC including seizure, post-traumatic hydrocephalus, and syndrome of trephined [4–7]. While cranioplasty is a routine and technically straightforward procedure, current data demonstrates failure and complication rates as high as 40% due to infection, hardware exposure, and autologous bone resorption [8–10]. As such, there has been a focus on shorter operating times, optimizing time between craniectomy and cranioplasty, and managing patient comorbidities to improve outcomes [11–13].

**Fig. 1:**
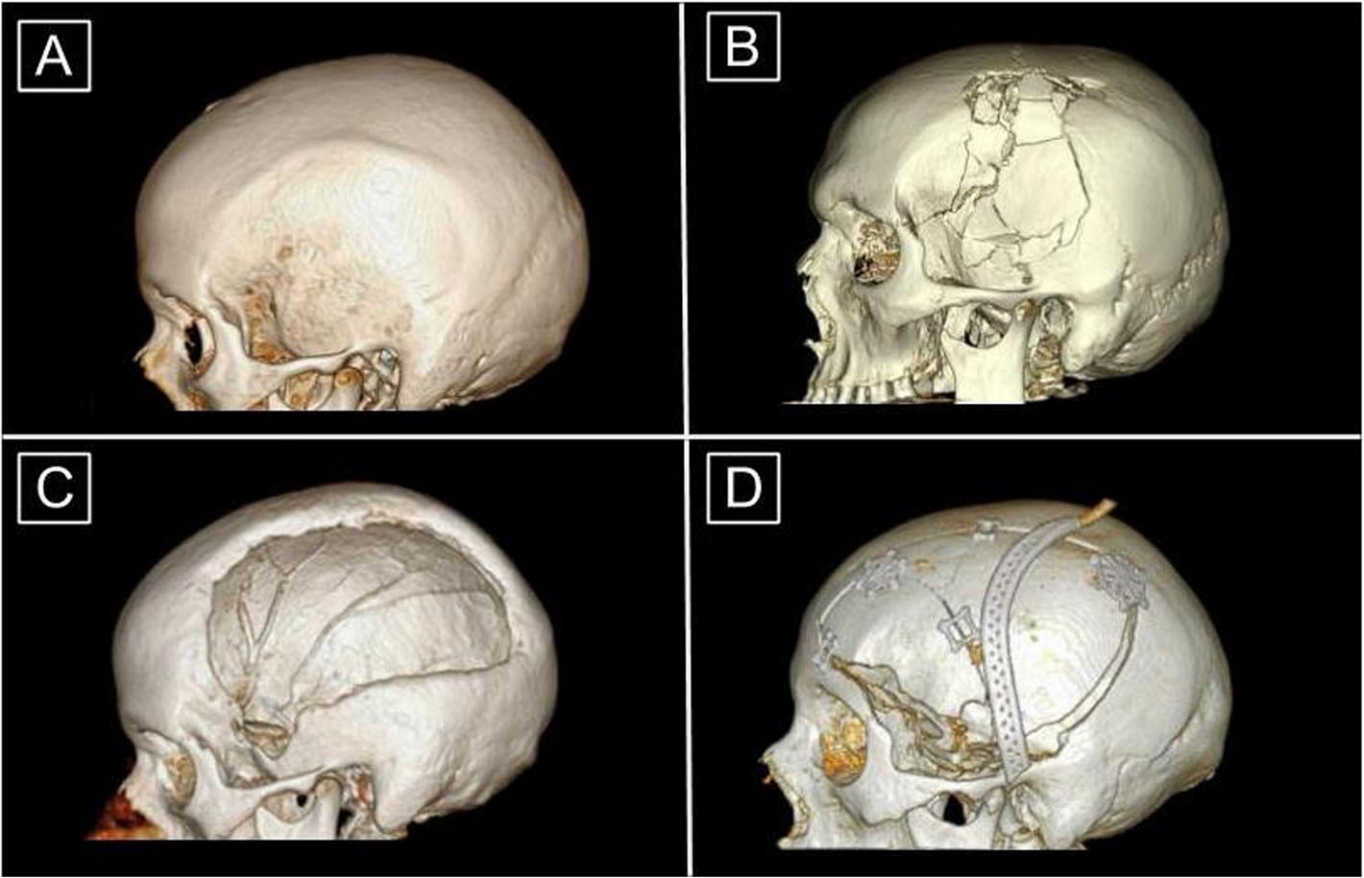
3D Reconstructions of Head CT Images in Craniectomy and Cranioplasty (**a**) Intact or normal skull. (**b**) Skull fracture that is overlying intracranial pathology requiring craniectomy. (**c**) Cranial defect after craniectomy. Note the asymmetric and heterogeneous nature of the outer border. (**d**) Post-operative subject who had undergone cranioplasty with autologous bone. The perforated piece overlying the skull is a Jackson-Pratt drain. Note that the bone fragments require extra structural support and do not fully repair the cranial defect along the inferior border

Another avenue that has been explored is the advancement in cranioplasty material and design. Traditionally, frozen or subcutaneously preserved autologous bone flaps have been used for reconstruction due to lower costs and anatomical fit [14, 15] (Fig. 1). However, current complication rates with this material, specifically bone resorption and infection, have highlighted the role for synthetic alternatives [16–21]. Currently, titanium mesh, polyetheretherketone (PEEK), polymethylmethacrylate (PMMA), and hydroxyapatite implants are being utilized. While each have their own added benefits, they also carry their own unique issues. Titanium mesh possesses noninflammatory and noncorrosive functionality while maintaining strength and malleability, yet creates imaging artifacts and carries an increased risk for metal hypersensitivity leading to device exposure [22–24]. PEEK demonstrates reduced complication and failure rates compared to autologous and titanium counterparts in limited studies [24, 25]. However, PEEK may not always incorporate with the native bone defect, leading to extrusion [23, 26]. PMMA implants have robust compression, heat and stress resistance, and strong adherence [23, 27], but have demonstrated greater infection rates and the potential for exothermic burn reaction due to a polymerization process [28, 29]. Lastly, hydroxyapatite is known for its contour ability and its excellent cosmesis, but lacks tensile strength and osseointegration, leaving the skull susceptible to fragmentation and infection [30–32]. Overall, synthetic implants have shown lower infection rates and absorption rates compared to autologous [23, 33, 34]. Yet, there are still issues to be addressed with these materials to maximize patient outcome.

**Fig. 2:**
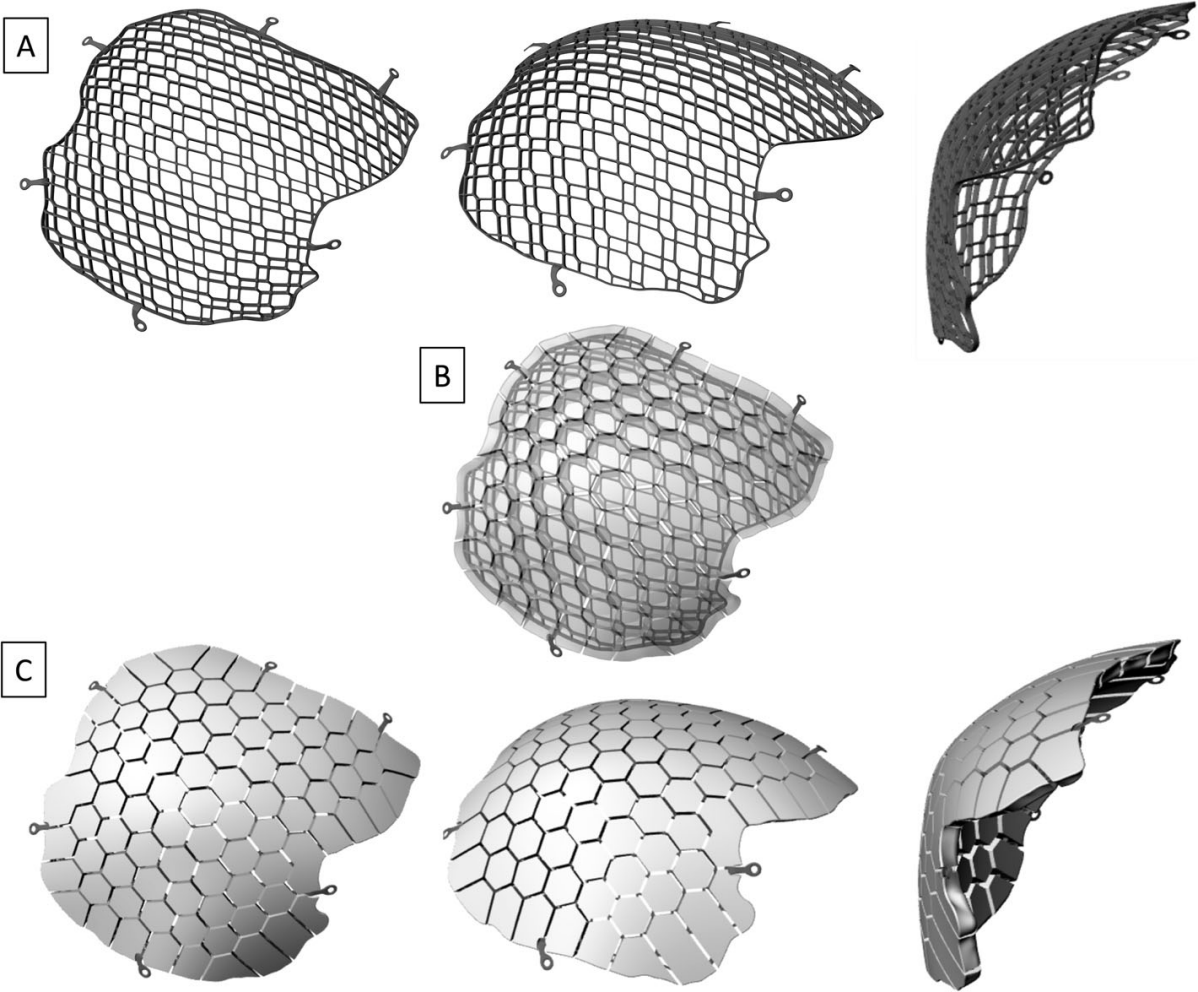
Images of 3D Printed Titanium Mesh Implants (**a**) Three views of the 3D printed titanium skeleton. (**b**) Transparent view of the 3D printed titanium skeleton embedded in the core of the calcium phosphate tiles. (**c**) Three views of the biocompatible calcium phosphate ceramic tiles situated upon the titanium skeleton layer. Note the mosaic tile design incorporating inter-tile spacing to permit fluid movement throughout the implant to enhance revascularization

A novel implant type, 3D-printed calcium phosphate cement mosaic tiles on a titanium mesh, has recently received Food and Drug Administration approval in the United States [35]. Along with its antimicrobial and osteogenic qualities, these 3D implants are also designed to minimize operation times and optimize cranial defect repair through individualized design (Fig. 2). Due to their recent emergence in the field, there has been limited data published on the failure rates of these 3D implants. One recently published study looked at the outcomes of 50 Swedish patients with the 3D implant, 32 (64%) of whom had a prior failure with autologous or alloplastic implants and subsequently received the 3D implant [35]. Overall, they demonstrated a 7.5% explantation rate for the 3D implants, as well as in situ bone regeneration and osseointegration [36]. While these findings suggest a significant reduction in cranioplasty failure rates with 3D implants, the study did not investigate or compare the raw failure rates of cohorts receiving different cranioplasty types, and rather only focused on those who received a 3D implant. It also did not directly explore the statistical mediators or complexities between patient cohorts who receive different cranioplasty implant type.

In the present study, we evaluated cranioplasty procedures performed at a level-1 trauma hospital in the United States, specifically focusing on the unique factors with the potential to affect outcomes in 3D-printed grafts, autologous grafts, and other synthetic allografts cohorts. We attempt to better describe the pertinent features of cranioplasty failure at an urban trauma center by capturing patient comorbidities, risk factors, and surgical considerations and contribute to the literature evaluating cranioplasty material.

## Materials and methods

### Subject selection and study population

A retrospective chart review was performed on all patients that underwent cranioplasty from January 1st, 2015 to December 31st, 2017 at our level-1 Trauma Center, Hennepin County Medical Center in Minneapolis, Minnesota. Those included in the study were patients with a prior craniectomy followed by at least one cranioplasty procedure within the aforementioned time interval. Those with insufficient cranioplasty data or documentation, cranioplasty performed at another hospital, and absence of prior craniectomy information were excluded. The study was approved by and conducted in full compliance with the Hennepin Healthcare Research Institute Institutional Review Board with number HSR#17–4309.

### Clinical data

Clinical data from the electronic medical records (EMR) was retrospectively collected for all patients that had undergone a cranioplasty procedure within the specified time interval, based on relevant ICD-9 and ICD-10 codes. Data was collected for each surgical admission including the initial craniectomy, primary cranioplasty, explantations, and subsequent cranioplasty to capture a holistic view of their neurosurgical history. We also collected information on patient demographics, comorbidities, underlying neurosurgical pathology, preoperative antibiotics, surgery length time, estimated blood loss (EBL), incision type, intraoperative vitals, postoperative complications, infection diagnosis and treatment, hospital length of stay, discharge destination, time between neurosurgical procedures, and mortality.

We defined cranioplasty failure as the need for explantation and surgical revision. Common indications included infection, fluid collection, bone resorption, and others. The time between the cranioplasty explantation was denoted as the time to failure. Additionally, we chose to evaluate each cranioplasty procedure separately, rather than evaluating each patient, such that if one patient underwent multiple cranioplasty procedures, each of those procedures was treated as independent of one another. If multiple procedures were performed during a single admission, the data was collected separately. Only the hospital admissions that included or involved a complication of a neurological surgery were included.

**Fig. 3:**
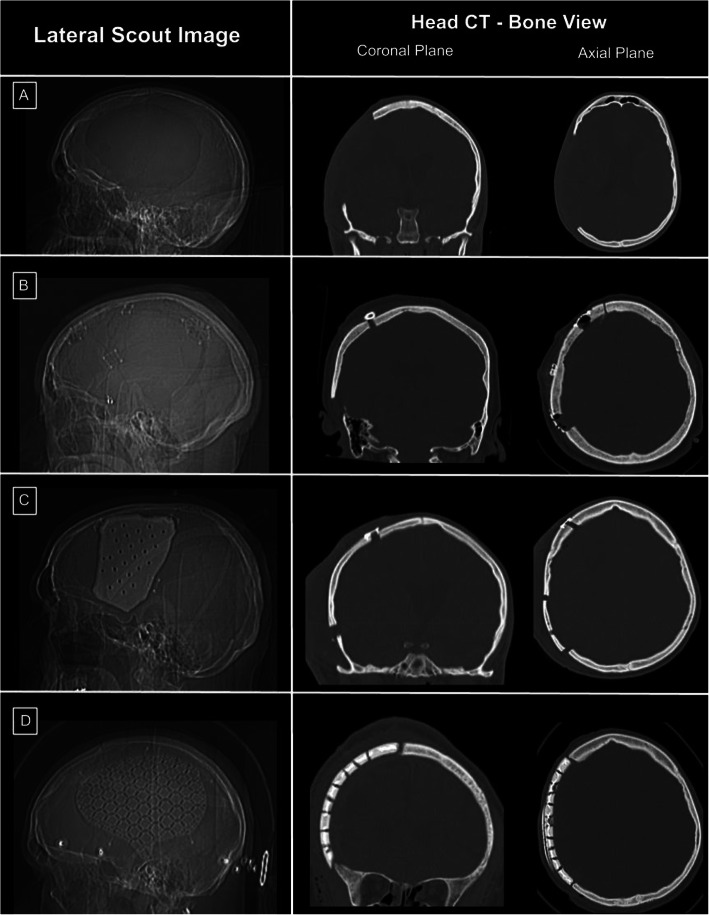
Head CT Images of Autologous, Synthetic, and 3D Printed Cranioplasty Implants (**a**) Images of a subject post-craniectomy. (**b**) Images of a subject post-cranioplasty with an autologous implant. Note the defect at the inferior border of the implant in the coronal plane. (**c**) Images of a subject post-cranioplasty with a synthetic implant. Note the improved profile of the skull border using the synthetic implant in the coronal plane as well as the remaining inferior defect. (**d**) Images of a subject post-cranioplasty with a 3D printed implant. Note the improved defect repair and restoration of the native skull border profile

Each cranioplasty procedure was classified into one of the following three groups: 3D-printed (OssDsign© – Uppsala, Sweden), autologous, and synthetic grafts (Fig. 3). The synthetic category included PEEK, titanium mesh, and PMMA implants. If a patient underwent multiple cranioplasty procedures, and if different implant materials were used for the separate procedures, then the procedure was grouped according to its respective implant category. An individual patient may have procedures separated into different groups, according to the type of implant material used.

### Statistical analysis

The cranioplasty procedures were categorized into three cohorts and compared. Continuous variables were expressed as the mean ± standard deviation, and categorical variables were expressed as a percentage or frequency.

## Results

### Sample demographics

In total, 60 patients underwent 71 total cranioplasty procedures. Our patient population was comprised of 40% females (*n* = 24) and 60% males (*n* = 40) with a mean age of 39.7 years (range 3–79). A majority of the subjects were Caucasian (63.3%), with the remainder being African American (16.7%), Hispanic (11.7%), and Other (8.3%). Insurance funding at the time of the cranioplasty was 45.0% private insurance, 33.3% medical assistance, and 20.0% Medicare/Medicaid. The comorbidity profile for all cohorts demonstrated a mean body mass index (BMI) of 25.9, a 20.0% incidence of hypertension, and a 13.3% incidence of Diabetes Mellitus Type II. Substance use was prevalent, with 53.3% of patients using alcohol, 36.7% using tobacco, and 20.0% using illicit drugs. Finally, a review of psychiatric diagnoses revealed 25.0% of subjects had depression, 18.3% had anxiety, 8.3% had Attention Deficit Hyperactivity Disorder (ADHD), and 13.3% had a different psychiatric condition (Table 1).

**Table 1:** Demographic Information

Characteristic	Cranioplasty
**Age**	
Mean (SD)	39.7 (17.9)
Range	3–79
**Sex**	
Male	36 (60%)
Female	24 (40%)
**Race**	
African American	10 (17%)
Caucasian	38 (63%)
Hispanic	7 (12%)
Other	5 (8%)
**Insurance**^a^	
Medicare/Medicaid	12 (20%)
Private Insurance	27 (45%)
Medical Assistance	20 (33%)
**Mechanism of Injury**	
Blunt Trauma	34 (57%)
Penetrating Trauma	5 (8%)
Spontaneous ICE	21 (35%)
**Pathology of Injury**	
Cerebral Edema	12 (20%)
Hemorrhage/Bleeds	38 (63%)
Penetrating Wound	10 (17%)
**BMI**	
Mean (SD)	25.9 (5.8)
**Substance History**	
Alcohol	32 (53%)
Tobacco	22 (37%)
Illicit Drugs	12 (20%)
**Psychiatric History**	
ADHD	5 (8%)
Anxiety	11 (18%)
Depression	15 (25%)
Other	8 (13%)
**Other Comorbidities**	
Diabetes Mellitus II	8 (13%)
Hypertension	12 (20%)

^a^Two patients had unknown insurance: one autologous and one synthetic

*ADHD* Attention deficit hyperactivity disorder, *BMI* Body mass index, *ICE* Intracranial event, *SD* Standard deviation

Small sample sizes did not allow for reliable statistical analysis

### Craniectomy data

The most common mechanisms of injury were blunt trauma (56.7%), spontaneous intracranial events (ICE) (35.0%) such as aneurysmal hemorrhage and strokes, and penetrating trauma (8.3%). The main underlying pathology being treated with primary craniectomy was hemorrhage (63.3%), followed by cerebral edema (20.0%) and penetrating cerebral wounds (16.7%).

### Implant data

Of these 71 unique cranioplasty procedures, 56 (78.9%) were a primary cranioplasty, 13 (18.3%) were a secondary cranioplasty, and two (2.8%) were a tertiary cranioplasty. Autologous bone flaps were utilized in 46 (64.8%) of these procedures: 44 primary, two secondary, and zero tertiary. 3D flaps were used for 13 (18.3%) procedures: six primary, six secondary, and one tertiary. Finally, synthetic flaps were used for 12 (16.9%) procedures: six primary, five secondary, and one tertiary. Of these synthetic flaps, eight were titanium, three were PEEK, and one was PMMA. See Fig. 2 for CT imaging of implant cohorts.

### Intraoperative metrics

The mean length of surgery for all procedures was 217.6 (± 69.3) minutes: 224.4 (± 77.3) minutes for autologous, 210.5 (± 47.9) minutes for synthetic, and 200.8 (± 54.3) minutes for 3D (Table 2). Two autologous and one synthetic outliers were removed from this analysis because they involved additional procedures (i.e. external carotid-internal carotid bypass, arteriovenous malformation resection, and tumor resection). The mean EBL for all procedures was 244.5 (± 155.9) mL: 257.6 (± 168.6) mL for autologous, 223.3 (± 132.3) mL for 3D, and 212.0 (± 125.1) mL for synthetic. There were also two autologous outliers with EBL > 1.5 L excluded, as one subject underwent a bilateral cranioplasty, and the other was a pediatric subject with extensive extracranial and intracranial hemorrhage.

**Table 2:** Descriptive Intraoperative Metrics for Cranioplasty

	Total	3D	Autologous	Synthetic
**Incision Type**				
Backwards ‘?’	48 (68%)	8 (17%)	31 (66%)	9 (19%)
T-Shaped	6 (8%)	2 (33%)	4 (67%)	0 (0%)
Linear	8 (11%)	1 (13%)	6 (75%)	1 (13%)
Trap Door	2 (3%)	0 (0%)	2 (100%)	0 (0%)
Other	7 (10%)	2 (29%)	3 (43%)	2 (29%)
**Surgery Length (min)**				
Mean	217.6	200.8	224.4	210.5
SD	69.3	54.3	77.3	47.9
**EBL (mL)**				
Mean	244.5	223.3	257.6	212.0
SD	155.9	132.3	168.6	125.1
**Time Since Craniectomy (d)**				
Mean	72.9	129.3	47.5	121.7
SD	64.3	52.6	41.8	92.2

‘?’: Question-mark, *EBL* Estimated blood loss, *SD* Standard deviation

Small sample sizes did not allow for reliable statistical analysis

### Incision type

For the type of surgical incision, a backwards question mark was used for 48 procedures (67.6%), linear for eight procedures (11.3%) T-shaped for six procedures (8.5%), trap door for two procedures (2.8%), and other types for seven procedures (9.9%) (Table 2). The incisions denoted as “other” included horseshoe, extended lacerations, and those without details in the EMR. Of note, all autologous cranioplasties utilizing a trap-door incision failed (*n* = 2), while none of the six using a linear incision failed. Additionally, three of the 31 autologous cranioplasties using a backwards question-mark incision failed (9.7%).

### Time since Craniectomy

The mean time interval between the craniectomy or explant to the cranioplasty was 72.9 (± 64.3) days overall. For the specific cohorts, the mean interval time was 47.5 (± 41.8) days for autologous, 129.3 (± 52.6) days for 3D, and 121.7 (± 92.2) days for synthetic implants. Three outliers were removed, one from the 3D group and two from the synthetic group, due to markedly prolonged time intervals that fell more than two standard deviations away from the mean. For analysis, the remaining 68 time intervals were classified into two groups: less than 3 months, and at least 3 months. In this study, the synthetic group had numerically more failures in patients whose time intervals were less than 3 months (*n* = 3, 60%) compared to those of at least 3 months (*n* = 0, 0%) (Table 2).

### Cranioplasty failure requiring reoperation

Out of the 71 total cranioplasty procedures, 14 (19.7%) required surgical revision (Table 3). These consisted of 11 primary cranioplasty failures (19.6% failure rate), two secondary failures, and one tertiary failure. Out of the 14 total failures, nine were autologous implants (64.3%), three were synthetic implants (21.4%), and two were 3D implants (14.3%). Within each category, three of the 12 total synthetic implants failed (25.0%), nine of the 46 total autologous implants failed (19.6%), and two of the 13 total 3D implants failed (15.4%).

**Table 3:** Cranioplasty Failure Rates

	Total	3D	Autologous	Synthetic
**Total Failures**				
Totals	14	2	9	3
% Failed	19.7%	15.4%	19.6%	25.0%
**Primary Implant**				
Totals	56	6	44	6
# Failed	11	1	8	2
% Failed	19.6%	16.7%	18.2%	33.3%
**Secondary/Tertiary Implant**				
Totals	15	7	2	6
# Failed	3	1	1	1
% Failed	20.0%	14.3%	50.0%	16.7%
**Reason for Revision**				
Fluid Collection	2	0	2	0
Infection	8	1	4	3
Bone Resorption	1	0	1	0
Other	3	1	2	0
**Time to Failure (d)**				
Mean	75.3	29.0	86.4	80.0
SD	87.9	8.5	94.6	114.1
**Follow-Up Time (d)**				
Mean	802.5	400.8	973.4	593.5
SD	340.0	106.4	276.0	217.1

*SD* Standard deviation

Small sample sizes did not allow for reliable statistical analysis

Collectively for the three groups, the average time to failure was 75.3 (± 87.9) days: the autologous group was 86.4 (± 94.6) days, 3D group was 29.0 (± 8.5) days, and synthetic group was 80.0 (± 114.14) days. Of note, two autologous grafts failed at 3713 and 2310 days, both of which required interval surgical revisions that did not meet our definition of failure (i.e. removal of a single piece of hardware without cranioplasty revision). They were both excluded in this analysis.

For the failures within the autologous subgroup, four patients needed revisions due to infection, three presented with fluid collection, one experienced bone resorption, and two had other pathologies. In the 3D subgroup, one presented with infection and one presented with wound failure. In the synthetic subgroup, all three presented with infection (Table 3).

In addition, within the autologous group, the comorbid factors of tobacco use (*n* = 6, 42.86%), and a pre-existing ADHD diagnosis (*n* = 3, 100%) were present most commonly in those that failed.

## Discussion

Complications from cranioplasty procedures continue to be an important issue despite the procedure’s routine nature. Prior work has investigated the feasibility of utilizing 3D technology for cranioplasty implants [37–41]. A mold fabrication system has been developed that constructs cranioplasty implants with higher cranial index symmetry – matching the cranial defect more accurately – than autologous types [41–44] (Figs. 1 and 2). Other research has assessed these 3D implants using cadavers as proxies, providing insight into the efficiency of the technique and the clinical applications of employing procedures of this genre [45]. The most promising research looked retrospectively at patients undergoing calcium phosphate-based implants with 3D-printed titanium mesh reinforcement [36]. Notably, the need for explantation was found in 7.5% of these patients, who collectively had a previous failure rate of 64% in prior autologous or alloplastic implants. This study, however, did not assess the failure rates between different cranioplasty types, nor did they delve into the factors contributing to these outcomes. While the results from the present study were based on a population size that did not allow for reliable statistical testing, the raw data does provide valuable insight into the promising potential of 3D-printed implants and the elements involved in reducing cranioplasty failures.

Our study demonstrated an overall cranioplasty failure rate of 19.7% at our institution. Notably, the failure rates of synthetic implants, autologous implants, and 3D implants were 25.0%, 19.6%, and 15.4%, respectively. Infection was the leading cause of failure, with infection rates of 25.0% for synthetic, 8.7% for autologous, and 7.7% for 3D. Of note, 3D implants in this study had shorter follow-up times on average (400.8 ± 106.4 days) compared to the other cohorts. This may favorably bias failure rates in the 3D cohort as complications can present after a prolonged period and is noted as a limitation.

**Fig. 4:**
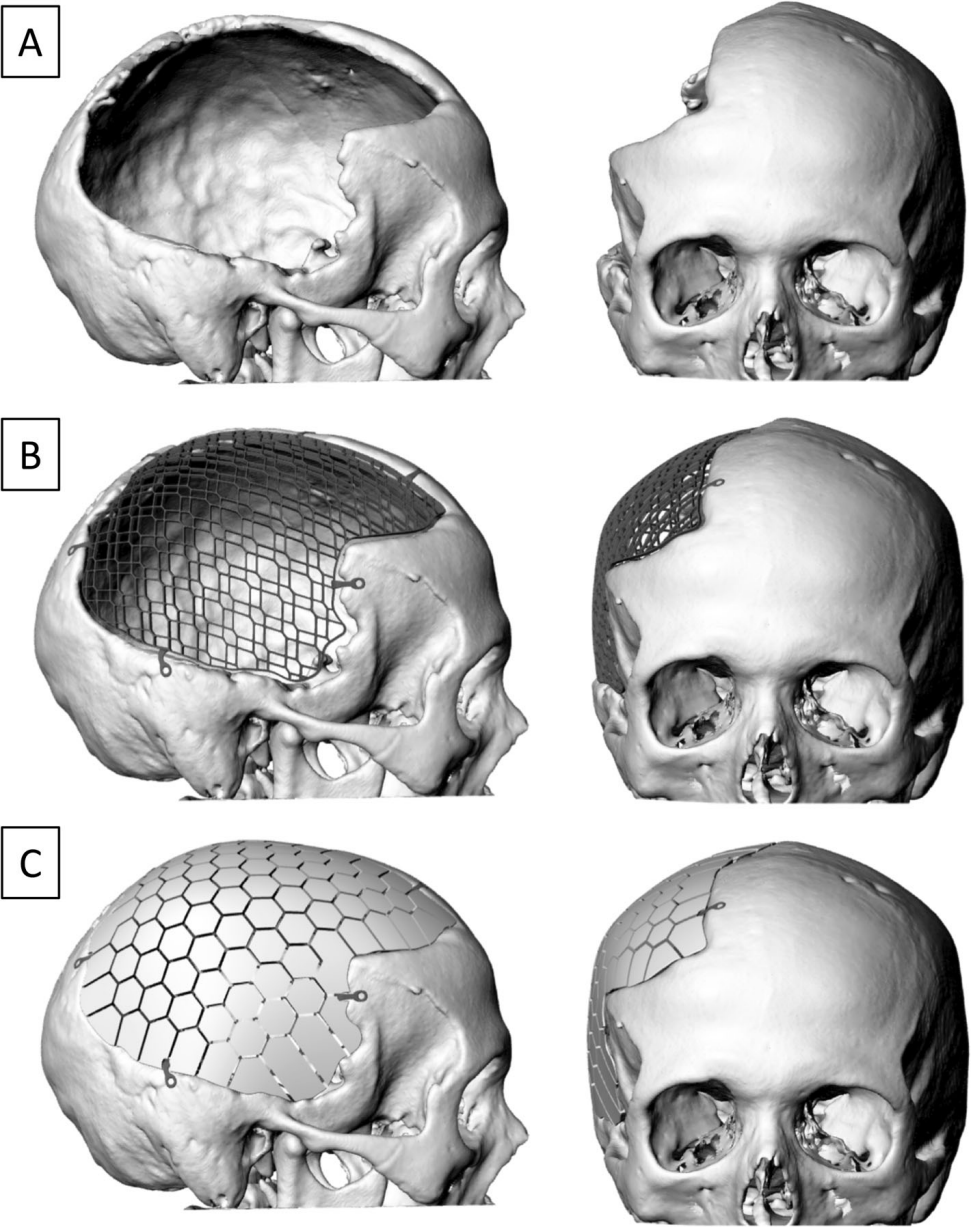
Images of 3D Reconstructions of Cranial Defect and Incorporated Implant (**a**) Cranial defect after craniectomy. (**b**) Titanium skeleton with its adjustable, pre-designed fixation arms filling in the cranial defect, allowing for patient-specific customization. (**c**) Calcium phosphate mosaic implant integrated into the underlying titanium reinforcement

There has been a recent focus on the choice of surgical incision used and its role in cranioplasty failure. Current theories suggest that optimizing flap vascularity improves healing thus limiting infection, hardware exposure, and need for reoperation. Our study found that both of the autologous implants placed using a trap door incision, which has suboptimal vascularity, failed (100%) compared to the backwards question mark (66%) and linear incisions (75%) for this cohort. This may support the hypothesis that an improved vascularity of a straighter incision may be beneficial for surgical outcomes. We also found that tobacco use was more prevalent in autologous failures (42.9%), and that all autologous cranioplasty patients with ADHD comorbidity resulted in failures. Time from the craniectomy or explant to the cranioplasty was also a factor in synthetic failure, with those implanted in less than 3 months after craniectomy failing 60% of the time, compared to those implants at least 3 months after craniectomy having a failure rate of 0%. Another intraoperative metric of note was surgery length; we found the shortest mean times for 3D implants (200.8 min), followed by synthetic (210.5 min) and autologous (224.4 min). While the differences in surgery lengths were not able to be statistically compared in our study due to small sample sizes, improvements in surgical efficiency and patient safety remains an important goal. The 3D-printed implants were designed precisely for this reason and to eliminate the need for manual intraoperative sculpting or reconstruction of fragmented skull pieces applied to alternative implant options (Fig. 4).

It should be noted that seven of 13 (53.8%) 3D cases, six of 12 (50.0%) synthetic cases, and two of 46 (4.3%) autologous cases were revisions of at least one previously failed cranioplasty procedure. With the current standard of care favoring the use of autologous bone flaps for primary cranioplasty, this is not unexpected but does add a level of complexity to the interpretation of our data. Considering that prior failure is a major risk factor for subsequent cranioplasty failure, the 3D and synthetic groups were already predisposed to complications. Interestingly, despite 46.2% of the 3D implants being utilized to replace a traditional implant that had failed, this cohort demonstrated the lowest failure rate (15.4%). Moreover, the only 3D procedure to be complicated by an identified infection was in a patient who had previously undergone an autologous cranioplasty that subsequently became infected, requiring a revision with the aforementioned 3D procedure. Perhaps, given the theoretical antimicrobial and intraoperative benefits of the 3D implant, larger studies may be able to explore the potential benefit of utilizing a 3D implant as the primary material to reduce the need to surgical revision.

As with any retrospective study, the interpretation of our data carries notable limitations. The first of these is variable duration of follow-up time in the different groups. The average time to failure was 75.3 days collectively, with the longest average being 86.4 days in the autologous group, and an average follow-up time of 802.5 days across all three groups (Table 3). For those patients whose cranioplasty procedure occurred more recently, it was ensured that their charts were assessed for a minimum of 6 months following the cranioplasty, to allow enough time to observe any complications. With this consideration, it was determined that all but one procedure had follow-up times that were more than two standard deviations from the average time to failure. This strongly suggests that the vast majority of complications or failures that could arise from any given cranioplasty procedure were in fact observed and recorded within the timeframe of the study. Nonetheless, the longer the graft is followed, the greater the likelihood of identifying a failure.

The potential impact of confounding variables on failure rates between implant types must also be mentioned. For example, patients with a lower BMI and a preference towards optimal incision type within an implant type may skew outcomes. Another notable limitation is that due to our small sample size, we were unable to perform valid statistical analysis to determine the significance of specific factors on cranioplasty outcomes. Nonetheless, the data captured here are informative. As there are not currently any U.S. studies that compare 3D-printed implants to autologous and synthetic implants, the expansion of the present study to multiple hospitals and the inclusion of additional 3D and synthetic implants could potentially produce more robust and constructive findings. Future studies would also benefit from longer patient follow-up at various intervals, examining both minor and major complications to add more depth and detail to the findings on cranioplasty failures.

## Conclusion

Based on the findings from the present study, 3D-printed implants demonstrate potentially favorable failure rates, infection rates, and surgery times compared to autologous and synthetic implants in TBI cranial reconstruction, although statistical significance could not be determined given our limited study population. Further research on the efficacy of 3D implants and their impact on surgical outcomes using larger sample sizes and longer follow-up assessments are warranted.

## Data Availability

All data generated during the current study are available from the corresponding author on reasonable request.

## Abbreviations

3D: Three-dimensional
ADHD: Attention deficit hyperactivity disorder
BMI: Body mass index
DC: Decompressive craniectomy
EBL: Estimated blood loss
EMR: Electronic medical records
ICE: Intracranial events
PEEK: Polyetheretherketone
PMMA: Polymethylmethacrylate
TBI: Traumatic brain injury

## References

[1] Kolias AG, Kirkpatrick PJ, Hutchinson PJ (2013). Decompressive craniectomy: past, present and future. Nat Rev Neurol.

[2] Servadei F, Compagnone C, Sahuquillo J (2007). The role of surgery in traumatic brain injury. Curr Opin Crit Care.

[3] Stocchetti N, Maas AIR (2014). Traumatic intracranial hypertension. N Engl J Med.

[4] Ashayeri K, Jackson EM, Huang J, Brem H, Gordon CR (2016). Syndrome of the trephined: a systematic review. Neurosurgery..

[5] Ban SP, Son YJ, Yang HJ, Chung YS, Lee SH, Han DH (2010). Analysis of complications following decompressive craniectomy for traumatic brain injury. J Korean Neurosurg Soc.

[6] Honeybul S, Ho KM (2016). Cranioplasty: morbidity and failure. Br J Neurosurg.

[7] Kurland DB, Khaladj-Ghom A, Stokum JA, Carusillo B, Karimy JK, Gerzanich V (2015). Complications associated with decompressive craniectomy: a systematic review. Neurocrit Care.

[8] Bobinski L, Koskinen LOD, Lindvall P (2013). Complications following cranioplasty using autologous bone or polymethylmethacrylate—retrospective experience from a single center. Clin Neurol Neurosurg.

[9] Coulter IC, Pesic-Smith JD, Cato-Addison WB, Khan SA, Thompson D, Jenkins AJ (2014). Routine but risky: a multi-Centre analysis of the outcomes of cranioplasty in the northeast of England. Acta Neurochir.

[10] Lee CH, Chung YS, Lee SH, Yang HJ, Son YJ (2012). Analysis of the factors influencing bone graft infection after cranioplasty. J Trauma Acute Care Surg.

[11] Kim H, Sung SO, Kim SJ, Kim SR, Park IS, Jo KW (2013). Analysis of the factors affecting graft infection after cranioplasty. Acta Neurochir.

[12] Piedra MP, Nemecek AN, Ragel BT (2014). Timing of cranioplasty after decompressive craniectomy for trauma. Surg Neurol Int.

[13] van de Vijfeijken SECM, Groot C, Ubbink DT, Vandertop WP, Depauw PRAM, Nout E (2019). Factors related to failure of autologous cranial reconstructions after decompressive craniectomy. J Cranio-Maxillofac Surg.

[14] Brommeland T, Rydning PN, Pripp AH, Helseth E (2015). Cranioplasty complications and risk factors associated with bone flap resorption. Scand J Trauma Resusc Emerg Med.

[15] Cabraja M, Klein M, Lehmann TN (2009). Long-term results following titanium cranioplasty of large skull defects. Neurosurg Focus.

[16] Cheah PP, Rosman AK, Cheang CK, Idris B (2017). Autologous cranioplasty post-operative surgical site infection: does it matter if the bone flaps were stored and handled differently?. Malays J Med Sci.

[17] Grant GA, Jolley M, Ellenbogen RG, Roberts TS, Gruss JR, Loeser JD (2004). Failure of autologous bone—assisted cranioplasty following decompressive craniectomy in children and adolescents. J Neurosurg Pediatr.

[18] Honeybul S, Morrison DA, Ho KM, Lind CRP, Geelhoed E (2017). A randomized controlled trial comparing autologous cranioplasty with custom-made titanium cranioplasty. J Neurosurg.

[19] Kim SH, Kang DS, Cheong JH, Kim JH, Song KY, Kong MH (2017). Comparison of complications following cranioplasty using a sterilized autologous bone flap or polymethyl methacrylate. Korean J Neurotrauma.

[20] Lethaus B, Bloebaum M, Essers B, ter Laak MP, Steiner T, Kessler P (2014). Patient-specific implants compared with stored bone grafts for patients with interval cranioplasty. J Craniofac Surg.

[21] Piitulainen JM, Kauko T, Aitasalo KMJ, Vuorinen V, Vallittu PK, Posti JP (2015). Outcomes of cranioplasty with synthetic materials and autologous bone grafts. World Neurosurg.

[22] Shah AM, Jung H, Skirboll S (2014). Materials used in cranioplasty: a history and analysis. Neurosurg Focus.

[23] Sun Y, Hu Y, Yuan Q, Yu J, Wu X, Du Z (2018). Association between metal hypersensitivity and implant failure in patients who underwent titanium cranioplasty. J Neurosurg.

[24] Thien A, King NKK, Ang BT, Wang E, Ng I (2015). Comparison of polyetheretherketone and titanium cranioplasty after decompressive craniectomy. World Neurosurg.

[25] Punchak M, Chung LK, Lagman C, Bui TT, Lazareff J, Rezzadeh K (2017). Outcomes following polyetheretherketone (PEEK) cranioplasty: systematic review and meta-analysis. J Clin Neurosci.

[26] Lethaus B, Safi Y, ter Laak-Poort M, Kloss-Brandstätter A, Banki F, Robbenmenke C (2012). Cranioplasty with customized titanium and PEEK implants in a mechanical stress model. J Neurotrauma.

[27] Marchac D, Greensmith A. Long-term experience with methylmethacrylate cranioplasty in craniofacial surgery. J Plast Reconstr Aesthet Surg. 2008;61:744–52. 10.1016/j.bjps.2007.10.064. 10.1016/j.bjps.2007.10.055 18474454

[28] Blum KS, Schneider SJ, Rosenthal AD. Methyl methacrylate cranioplasty in children: long-term results. PNE. 1997;26:33–5. 10.1159/000121158. 10.1159/000121158 9361115

[29] Matsuno A, Tanaka H, Iwamuro H, Takanashi S, Miyawaki S, Nakashima M, et al. Analyses of the factors influencing bone graft infection after delayed cranioplasty. Acta Neurochir (Wien). 2006;148:535–40. 10.1007/s00701-006-0740-6. 10.1007/s00701-006-0740-6 16467959

[30] de Bassumpção M, Fonoff ET, Teixeira MJ. Early resorption of an artificial bone graft made of calcium phosphate for cranioplasty: case report. Neuropsychiatr Dis Treat. 2013;9:1801–2. 10.2147/ndt.s43806. 10.2147/NDT.S43806 PMC3833462 24265553

[31] Gilardino MS, Cabiling DS, Bartlett SP. Long-term follow-up experience with carbonated calcium phosphate cement (Norian) for cranioplasty in children and adults. Plastic Reconstr Surg. 2009;123:983–94. 10.1097/prs.0b013e318199f6ad. 10.1097/PRS.0b013e318199f6ad 19319064

[32] Kumar NG, Sudeep S, Balwan R. Cranioplasty of hemispherical defects using calcium phosphate cements along with titanium mesh: our experience. J Maxillofac Oral Surg. 2015;14:920–4. 10.1007/s12663-015-0776-3. 10.1007/s12663-015-0776-3 PMC4648777 26604464

[33] van de Vijfeijken SECM, Münker TJAG, Spijker R, Karssemakers LHE, Vandertop WP, Becking AG, et al. Autologous bone is inferior to alloplastic cranioplasties: safety of autograft and allograft materials for cranioplasties, a systematic review. World Neurosurg. 2018;117:443–52.e8. 10.1016/j.wneu.2018.05.193. 10.1016/j.wneu.2018.05.193 29879511

[34] Zanaty M, Chalouhi N, Starke RM, Clark SW, Bovenzi CD, Saigh M, et al. Complications following cranioplasty: incidence and predictors in 348 cases. J Neurosurg. 2015;123:182–8. 10.3171/2014.9.jns14405. 10.3171/2014.9.JNS14405 25768830

[35] Bonda DJ, Manjila S, Selman WR, Dean D. The recent revolution in the design and manufacture of cranial implants: modern advancements and future directions. Neurosurgery. 2015;77:814–24. 10.1227/neu.0000000000000899. 10.1227/NEU.0000000000000899 PMC4615389 26171578

[36] Kihlström Burenstam Linder L, Birgersson U, Lundgren K, Illies C, Engstrand T. Patient-specific titanium-reinforced calcium phosphate implant for the repair and healing of complex cranial defects. World Neurosurg. 2019;122:e399–407. 10.1016/j.wneu.2018.10.061. 10.1016/j.wneu.2018.10.061 30342265

[37] Abdel Hay J, Smayra T, Moussa R. Customized polymethylmethacrylate cranioplasty implants using 3-dimensional printed polylactic acid molds: technical note with 2 illustrative cases. World Neurosurg. 2017;105:971–9.e1. 10.1016/j.wneu.2017.05.007. 10.1016/j.wneu.2017.05.007 28502686

[38] Kim BJ, Hong KS, Park KJ, Park DH, Chung YG, Kang SH. Customized cranioplasty implants using three-dimensional printers and polymethyl-methacrylate casting. J Korean Neurosurg Soc. 2012;52:541–6. 10.3340/jkns.2012.52.6.541. 10.3340/jkns.2012.52.6.541 PMC3550422 23346326

[39] Mobbs RJ, Coughlan M, Thompson R, Sutterlin CE, Phan K. The utility of 3D printing for surgical planning and patient-specific implant design for complex spinal pathologies: case report. J Neurosurg Spine. 2017;26:513–8. 10.3171/2016.9.spine16371. 10.3171/2016.9.SPINE16371 28106524

[40] Parthasarathy J. 3D modeling, custom implants and its future perspectives in craniofacial surgery. Ann Maxillofac Surg. 2014;4:9–18. 10.4103/2231-0746.133065. 10.4103/2231-0746.133065 PMC4073471 24987592

[41] Tan ETW, Ling JM, Dinesh SK. The feasibility of producing patient-specific acrylic cranioplasty implants with a low-cost 3D printer. J Neurosurg. 2016;124:1531–7. 10.3171/2015.5.jns15119. 10.3171/2015.5.JNS15119 26566203

[42] Lim JY, Kim N, Park JC, Yoo SK, Shin DA, Shim KW. Exploring for the optimal structural design for the 3D-printing technology for cranial reconstruction: a biomechanical and histological study comparison of solid vs. porous structure. Childs Nerv Syst. 2017;33:1553–62. 10.1007/s00381-017-3486-y. 10.1007/s00381-017-3486-y 28623521

[43] Msallem B, Beiglboeck F, Honigmann P, Jaquiéry C, Thieringer F. Craniofacial reconstruction by a cost-efficient template-based process using 3D printing. Plast Reconstr Surg Glob Open. 2017;5:e158. 10.1097/gox.0000000000001582. 10.1097/GOX.0000000000001582 PMC5732683 29263977

[44] Oh J. Recent advances in the reconstruction of cranio-maxillofacial defects using computer-aided design/computer-aided manufacturing. Maxillofac Plast Reconstr Surg. 2018;40:2. 10.1186/s40902-018-0141-9. 10.1186/s40902-018-0141-9 PMC5797724 29430438

[45] Evins AI, Dutton J, Imam SS, Dadi AO, Xu T, Cheng D, et al. On-demand intraoperative 3-dimensional printing of custom cranioplastic prostheses. Oper Neurosurg (Hagerstown). 2018;15:341–9. 10.1093/ons/opx280. 10.1093/ons/opx280 PMC6887998 29346608

